# Fostering resilience and well-being in emerging adults with adverse childhood experiences: study protocol for a randomized controlled trial to evaluate the FACE self-help app

**DOI:** 10.1186/s40359-024-01560-9

**Published:** 2024-02-19

**Authors:** Jeannette Brodbeck, Salome I.R. Bötschi, Neela Vetsch, Lina Stallmann, Johanna Löchner, Thomas Berger, Stefanie J. Schmidt, Simon Marmet

**Affiliations:** 1 School of Social Work, University of Applied Sciences and Arts Northwestern Switzerland, Riggenbachstrasse 16, CH-4600 Olten, Switzerland; 2 Department of Clinical Psychology, University of Bern, Fabrikstrasse 8, CH-3012 Bern, Switzerland; 3 Swiss Center for Affective Science, University of Geneva, Chemin des Mines 9, CH-1202 Geneva, Switzerland; 4 Department of Child and Adolescent Psychiatry, Psychosomatics and Psychotherapy, University Hospital of Psychiatry and Psychotherapy, Tuebingen, Germany; 5 Department of Clinical Child and Adolescent Psychology, University of Bern, Fabrikstrasse 8, CH-3012 Bern, Switzerland

**Keywords:** Adverse childhood experiences, Child maltreatment, E-health, Resilience, Emotion regulation, Social information processing, Emerging adulthood, RCT

## Abstract

**Background:**

Adverse childhood experiences (ACE) are linked to an increased risk of psychological disorders and lower psychosocial functioning throughout life. This study aims to evaluate the FACE self-help app, designed to promote resilience and well-being in emerging adults with a history of ACE. The app is based on cognitive-behavioural principles and consists of two thematic components: (1) self- and emotion regulation (SER) and (2) social skills and biases in social information processing (SSIP).

**Methods:**

The efficacy of the app will be tested through a single-centre, two-arm randomized controlled trial, comparing an active intervention group against a waiting list control group. The active group is divided into two subgroups, in which the two components are delivered in a different order to investigate differential effects in a crossover design. Up to 250 emerging adults aged 18 to 25 years with a history of ACE from a general population cohort study will be recruited. The primary objective is to test the efficacy of the app in improving resilience (primary outcome) and well-being (co-primary outcome) compared to a waiting list control group and to examine the stability of these effects. The secondary objectives include testing the efficacy of the app in improving the secondary outcomes, i.e., self-efficacy in managing emotions, problem solving, fear of evaluation, social avoidance, and self-esteem; examining the differential effects of the two components; and assessing the effect of the app on real-life data on resilience, affective states, distress in social interactions and coping strategies. Furthermore, the study will investigate potential moderators (e.g. ACE severity) and mediators of intervention outcomes (e.g. self-efficacy in managing emotions).

**Discussion:**

The results will provide insights into the efficacy of the self-help intervention as well as mediators and moderators of outcomes. Furthermore, results will extend the existing knowledge by testing the differential effects of the SER and SSIP component on the outcomes. Findings can inform improvements to the FACE app and the development of other interventions for this target group and assess its potential as a scalable, low-threshold intervention to support emerging adults with a history of ACE in their transition to adulthood. Trial registration number: NCT05824182.

## Background

One out of three children worldwide experiences adverse childhood experiences (ACE) such as emotional, physical or sexual abuse, emotional or physical neglect or exposure to intimate partner violence [1]. It is well established that ACE have detrimental effects on mental and physical health throughout the lifespan. Therefore, primary prevention of the occurrence of ACE and early interventions in childhood are paramount. However, there are limitations to these approaches. Preventive interventions generally require the collaboration of parents or legal guardians, which cannot always be expected. Furthermore, meta-analyses have yielded conflicting results regarding the efficacy of prevention targeted at parents [2, 3].

Later in life, the transition from adolescence to young adulthood offers a window of opportunity for selective prevention directly targeting individuals affected by ACE. Emerging adults are better able to distance themselves from an adverse family environment and have more control of their own lives [4]. Multiple transitions, such as entering new stages of professional or educational life, moving away from one’s parents and shifting from economic dependence to independence, offer turning points and opportunities for redirecting maladaptive trajectories into healthier paths [5]. At the same time, this stage in life also holds a risk of the consolidation of problems, deterioration of trajectories or the onset of destructive outcomes, such as substance use, sexual risk behaviour, unemployment, deliberate self-harm and suicide [6–8]. Interventions at this stage in life are thus likely to have a large impact and offer high potential for preventing the need for more intensive psychotherapy for later chronic dysfunctions. Still, this critical phase is often accompanied by a gap in health and social service delivery for emerging adults: as they are no longer supposed to use services for children and adolescents, a change to the adult health service system might not appropriately address the needs of vulnerable emerging adults [9]. Additionally, the gap between the need for and the provision of care is assumed to be especially large for children, families and adults with dysfunctions associated with traumatic experiences and ACE [10, 11].

Digital self-help interventions (e-health) or interventions delivered on smartphones (mobile- or m-health) may bridge the resulting gap, as they are scalable, affordable and a convenient, user-friendly, low-threshold, anonymous and immediate way of delivering interventions in daily life [12]. Digital self-help and m-health interventions have been shown to improve resilience, well-being and emotion regulation and decrease symptoms of distress, depression, and anxiety [13–17]. Nevertheless, the effects were often only small to moderate; many m-health interventions lack a theoretical basis and additional studies with general population samples are needed [13, 16]. Moreover, digital interventions often result in low engagement, high attrition, and substantial dropout [18, 19]. Thus, improving users’ acceptance of the digital intervention is highly relevant for ensuring long-term usage. Nevertheless, a study suggested that more than 70% of adolescents would use an e-health intervention and that 32% would prefer an e-health intervention over traditional face-to-face support [20].

Fostering resilience, self- and emotion regulation as well as social information processing can present meaningful transdiagnostic targets for e- or m-health interventions. Resilience, defined as the ability of a person to function successfully despite aversive experiences and to adapt to a difficult situation, is a key protective factor against the negative health consequences of ACE [21, 22] and may thwart the development of mental health problems [23]. Pronounced resilience protects against negative consequences, while the experience of ACE is generally associated with lower resilience [23, 24]. Thus, to minimize negative effects on health, it is essential to promote the resilience of people affected by ACE through appropriate interventions [24].

Furthermore, self- and emotion regulation are crucial targets for interventions for emerging adults with a history of ACE. Emotion regulation has been conceptualized as part of the broader concept of self-regulation. The self-regulation promotion model identifies behavioural skills (e.g. stress management, impulse control, or goal setting and planning), cognitive skills (e.g. problem-solving, self-reflection, appraisal/reappraisal) and emotional skills (e.g. recognizing cues for distress, self-calming strategies, or acceptance of emotions) as targets for promoting self-regulation [25]. For the developmental stage of emerging adulthood in general, Murray and colleagues suggested providing guidance for complex problem solving and support for coping with significant stressors [25]. Emerging adults with a history of ACE may benefit especially from such interventions: ACE were associated with less adaptive emotion regulation [26, 27] and dysfunctional emotion regulation was found to mediate the association between ACE and mental and physical health problems [28–30]. Moreover, emotion regulation skills also affect the resilience of a person [31–33]. ACE were also associated with decreased problem-solving and coping skills [27]. Thus, improving self- and emotion regulation strategies seems to be a promising strategy for mitigating the negative consequences of ACE [28].

Related to self- and emotion regulation, self-esteem was found to be a mediator of the association between emotion regulation strategies and resilience among emerging adults [34]. ACE correlated negatively with self-esteem [35]. Low self-esteem, in turn, has a negative impact on mental health [36] and there is a consistent link between self-esteem and psychopathology [37]. Self-esteem has been shown to be a mediator between ACE and depression, loneliness, perceived stress and life stress [38], between child maltreatment and depressive symptoms [39] and between child maltreatment and well-being [40].

In addition to self- and emotion regulation, social skills and social information processing are important factors for fostering resilience and coping with ACE. ACE were associated with less adaptive social cognition and biased social information processing [41, 42]. Moreover, lower social support and loneliness have been established as a mechanisms linking ACE to adolescent or adult well-being and psychopathology [43–48].

### The present study

The FACE study focuses on self- and emotion regulation and social information processing as links between ACE and psychosocial functioning and as key components for fostering resilience. These components are in line with the transdiagnostic model of mechanisms linking childhood trauma to psychopathology that describes social information processing (enhanced threat detection and hostile attribution bias) and emotion processing (heightened emotion reactivity and poor emotion regulation) as malleable mechanisms linking childhood trauma to internalising and externalising psychopathology and that social support can act as a protective factor [49]. While there are many other factors that can influence the consequences of ACE such as biological, cognitive, or environmental factors, self- and emotion regulation and social information processing have consistently been associated with poorer mental health and there are several evidence-based interventions that target these factors.

The FACE study consists of two interlinked subprojects: (1) a three-wave epidemiological study to investigate emotion regulation and social information processing as mechanisms that link ACE with psychosocial functioning in emerging adulthood [50] and (2) the FACE intervention study, which is presented in this study protocol. The development of a specific transdiagnostic intervention for emerging adults with a history of ACE is important for several reasons: Participants may benefit from specific information about ACE and ACE-related content to develop a better understanding of their experiences. We also expect that a specific intervention addressing frequent questions, problems and needs of emerging adults with a history of ACE leads to higher enrolment, adherence, and efficacy of the intervention. Moreover, emerging adults with a history of ACE present a wide range of clinical and subthreshold symptoms including depressive, anxious and Posttraumatic Stress Disorder symptoms which suggests a transdiagnostic approach for interventions. Traditional e-health interventions are often designed for specific disorders and require the presence of this clinical diagnosis as inclusion criteria. The FACE intervention study is the first clinical trial of a specific m-health intervention targeted at emerging adults with a history of ACE and explores moderators and mediators of the intervention outcome. It not only uses retrospective self-report questionnaires to evaluate the intervention, but also ecological momentary assessments (EMA) to collect repeated real-time data of affective states and social interactions in a natural environment. Thus, the FACE study extends the existing knowledge on self- and emotion regulation and social information processing as mechanisms for fostering resilience in emerging adulthood.

## Methods

### Objectives

**Fig. 1:**
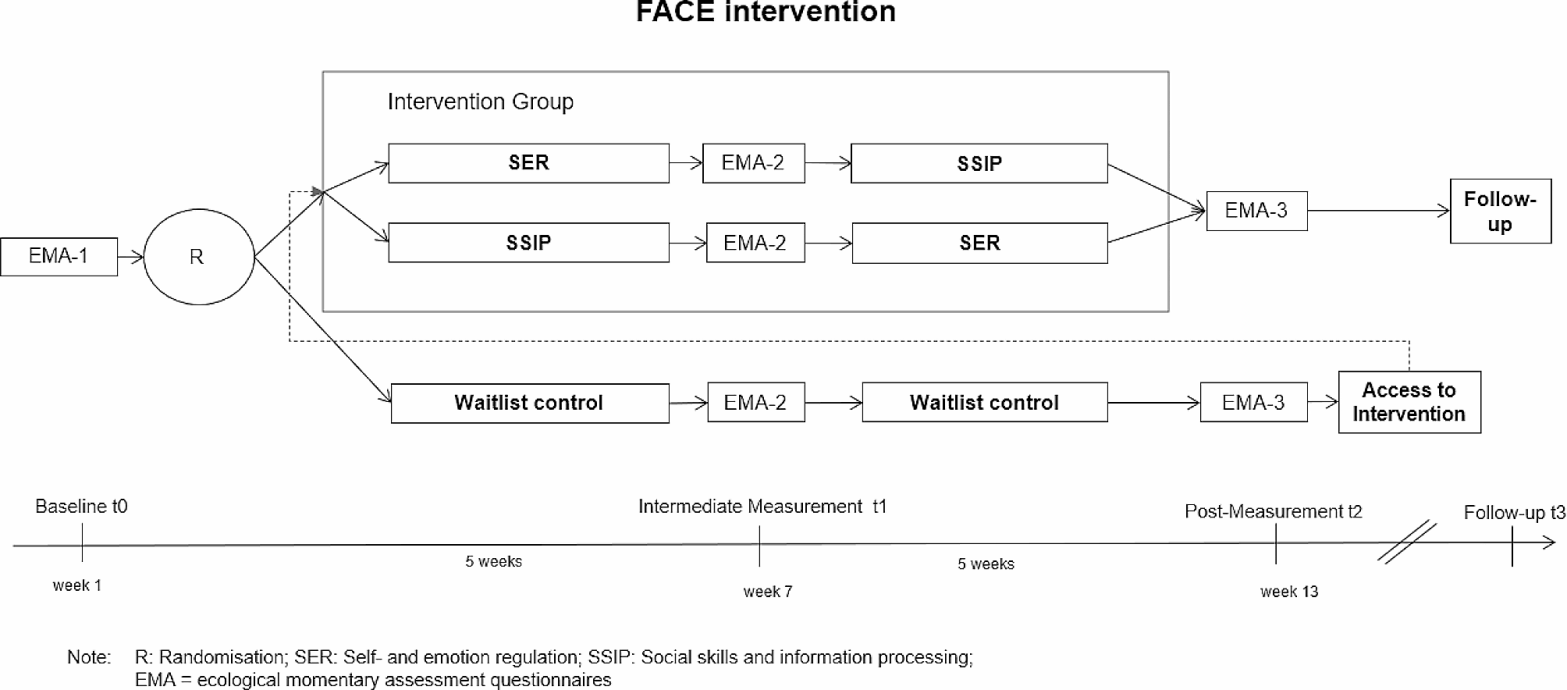
Overview of the study design

The primary objective is to test the efficacy of the FACE self-help app in improving resilience (primary outcome) and well-being (co-primary outcome) in emerging adults with a history of ACE compared to a waiting list control group (see Fig. 1). Then we aim to test the stability of the effects across three months in the intervention group. We hypothesize that the intervention group will significantly improve in all outcomes compared to the waiting list control group and that these outcomes will be stable.

### Secondary objectives

The secondary goals of this study are.

1) to test the efficacy of the FACE self-help app in improving self-efficacy for managing emotions and problem-solving as facets of self- and emotion regulation, fear of negative evaluation and social avoidance as facets of social skills and social information processing, as well as self-esteem (secondary outcomes).

2) to test the differential effects of the self- and emotion regulation (SER) and the social skills and social information processing (SSIP) components on all outcomes. We hypothesize that SER will specifically improve self-efficacy for managing emotions and problem-solving and thus will have a greater effect on these variables than will SSIP. Similarly, we expect that SSIP will specifically improve fear of negative evaluation and social avoidance and thus will have a greater effect on these variables than will SER.

3) We aim to analyse the effects of the FACE self-help app on real-life ecological momentary assessment data in affective states, distress in several areas of life, social interactions, the use of coping strategies and resilience. We expect that the FACE self-help app will improve these variables compared to the waiting list control group.

4) Finally, we assess the degree of satisfaction of the participants with the FACE self-help app.

### Exploratory objectives

In exploratory analyses, we will examine whether improvements in self-efficacy for managing emotions, problem-solving, or self-esteem are mediators of resilience and well-being outcomes. Furthermore, we test whether the sequence of the components affects the outcomes. Adaptive emotion regulation may promote learning from social interactions, as well as foster social flexibility and effective adaptation to different social environments, as intensive negative emotions may impede its use in distressing situations. Thus, the sequence of SER and SSIP might lead to better outcomes than the sequence of SSIP SER. Finally, we will investigate whether possible effects of the intervention are moderated e.g. by the number, type, or the severity of ACE.

### Trial design

The study is conceptualized as a single-centre randomized controlled trial comparing an active intervention group against a waiting list control group (see Fig. 1). The intervention consists of two components (SER and SSIP) which are further compared in a crossover design to examine the differential effects of the components. The allocation ratio of the number of participants in the control group to the number of participants in the intervention group is 1:2. Randomisation is performed using a computer-generated allocation sequence.

### Participants and procedures

Participants will be recruited between August 2023 and July 2024 from the FACE epidemiological study sample, which is based on a random list-based general population sample of emerging adults aged 18–21 at baseline in German-speaking Switzerland. This part of the study was described in detail in an earlier study protocol [50]. The FACE epidemiological study assesses different types of ACE, i.e., emotional abuse and neglect, physical abuse and neglect, sexual abuse, witnessing domestic violence, household dysfunctions but also peer verbal, physical and sexual abuse.

Emerging adults who reported a history of ACE in the FACE epidemiological study will be invited to participants in the self-help app evaluation study via an e-mail from the study collaborators. The e-mail invitation contains a written study information regarding the procedure and study content as well as a link to schedule an appointment for the interview. In the interview, the suitability of the self-help app for the situation of the respective emerging adult will be discussed and, in case of an acute crisis, participants will be informed about support and health-care services such as psychiatric outpatient clinics. After sending back the signed written informed consent to the study team, participants will receive the online questionnaires.

The inclusion criteria are (1) self-reported ACE; (2) written informed consent; (3) the possession of a smartphone; (4) mastery of the German language; (5) an age between 18 and 25 years. The exclusion criteria are (1) acute suicidality and (2) the inability to follow the procedures of the study, e.g. due to comprehension problems, visual impairment, lack of sufficient motor skills, or severe psychological or somatic disorders that require immediate treatment that impedes continuous work on the self-help program. These points will be discussed with the participants during the screening procedures.

### Measures

#### Primary and secondary outcomes

All primary and secondary outcomes will be measured via online self-report questionnaires. Participants will be asked to complete them before the intervention (t0), 5 weeks after starting the intervention (t1, intermediate measurement), which will correspond to the time needed to complete the first component, and 11 weeks after starting the intervention (t2). By this time, participants will have completed both components of the intervention, which corresponds to the post-measurement. Furthermore, participants will be asked to complete the questionnaire again 11 weeks after finishing the FACE self-help app, which serves as a follow-up (t4). An exception is the adapted Patient Satisfaction Questionnaire, which will be assessed only after the finishing first component (t1), and after finishing the second component of the self-help app (t2). Reminders to fill out the questionnaire will be sent after 2 and 4 weeks.

***Resilience*** will be assessed with the 10-item German version [51] of the Connor-Davidson Resilience Scale (CD-RISC) [21], which has been shown to be a valid and reliable measurement [51, 52]. The CD-RISC is widely used and has been shown to be sensitive to change following interventions [21, 53]. The items are rated on a 5-point scale ranging from 0– Not true at all to 4– True nearly all the time.

***Well-being*** will be assessed with the Warwick-Edinburgh Mental Well-being Scale (WEMWBS) [54]. The German version of the scale shows good reliability and validity [55]. The scale contains 14 items that are rated on a 5-point scale ranging from 1– never to 4– always.

***Self-efficacy for managing emotions*** will be assessed with the PROMIS Short Form v1.0 - Self-Efficacy for Managing Emotions [56]. PROMIS items were developed for use in the general population for different aspects of mental health. Method and assessment timepoint: The scale contains 7 items that are rated on a 5-point scale ranging from 1– never to 4– always.

***Fear of negative evaluation*** will be assessed with the German short version of the Fear of Negative Evaluation Scale (SANB-5) [57]. The scale contains 5 items that are rated on a 4-point Likert scale from 1 - almost never true to 4- almost always true.

***Social avoidance*** will be assessed with the subscale social-behavioural avoidance from the German version of the cognitive-behavioural avoidance scale (KBVS) [58]. The subscale contains 8 items that are rated on a 5-point Likert scale from 1 - not at all applicable for me to 5 - absolutely applicable for me.

***Problem-solving*** will be assessed with German version [59] of the short form of the Social Problem-Solving Inventory-Revised (SPSI-R) [60]. The scale contains 25 items that are rated on a 5-point scale from 0– not at all true to 4– extremely true.

***Self-esteem*** will be assessed with the German version [61] of the Rosenberg Self-Esteem Scale [62]. The scale contains 10 items that are rated on a 4-point scale from 1– not at all true to 4– extremely true.

***Acceptance*** of the intervention will be assessed with the Patient Satisfaction Questionnaire (ZUF-8), a self‐report measure that explores patients’ overall satisfaction with digital interventions [63]. It contains eight items that are rated on a 4‐point scale. The German version of the ZUF‐8 showed adequate psychometric properties with good internal consistency, construct validity, and concurrent validity [63].

#### Ecological momentary assessments

The self-report questionnaires described above assessing longer-lasting characteristics will be complemented with ecological momentary assessments (EMA) intended to measure real-life short-term states. For one week before the intervention (t0), one week after the first component (t1) and one week after the second component (t2), respectively, participants will be asked to complete a series of short online questionnaires, i.e., as many instances of the series as possible for them, as they receive 6 random prompts per day for 7 days. With each prompt, the assessment is available in a time window of 30 min and set to take 2–3 min to complete. Apart from the scale for momentary affect, the included items were developed based on conceptual considerations. All the items are rated on a 7-point scale from 0– not at all to 6– extremely.

*Momentary affect* will be assessed with an abbreviated version of the Positive And -Negative Affect Scale (PANAS) following previous research, that used similar EMA items [64, 65]. It contains 12 items that measure different affective states, for example, “At the moment I feel relaxed.”.

*Coping*: Five items assess coping based on previous research [66]. They measure different emotion regulation and coping strategies such as reappraisal, avoidance or help seeking, for example: “I tried to seek advice or comfort from others.”

##### Momentary burden

Seven items assess the level of distress in different areas of life (family/friends, thoughts/feelings, work/school, memories, leisure, others, and overall burden) in line with the questionnaire in the epidemiological study [50].

##### Resilience

One item assesses how well participants can cope with their current situation.

##### Social interactions

Nine items explore distress in social relationships, whether the participant has had social contact since the last assessment, and for the last significant social situation, the participant’s emotional state, e.g. “In the last social situation, I felt relaxed”. If someone did not have social contact, the reasons were assessed, e.g. avoidance of social contact or preferring to be alone.

#### Usage data and data from the FACE epidemiological study

Participants’ use of the FACE self-help app (logins, number of visits and time spent on modules/submodules pages) will be recorded as a measure of adherence. Furthermore, the data assessed in the FACE intervention study will be linked to the data assessed in the FACE epidemiological study, which included, among other variables, ACE and sociodemographic variables.

### Study intervention

The FACE app is based on a transdiagnostic model of mechanisms linking childhood trauma to psychopathology and addresses the thematic components Self- and Emotion Regulation (SER) and Social Skills and Social Information Processing (SSIP) for the promotion of resilience and well-being. It was developed based on well-established cognitive-behavioural principles that have been used in other web-based self-help apps [13] and offers psychoeducative materials and exercises in various formats (text, audio, video).

In addition to two components SER and SSIP (see Fig. 1), there are two additional parts intended to frame the user’s experience: an introduction and a conclusion/farewell. First, the general introduction, available to everyone from the start, explains the app interface, presents general information on ACE (psychoeducation about prevalence, types, and consequences), and encourages reflection on one’s personal status and resources plus the formulation of personal goals for the app. Towards the end of the intervention, the conclusion/farewell encourages the revisiting of the goals and ideas of the start and the reflection on what was most helpful while using the app. In addition, a collection of short activities and exercises relevant to everyday life and promoting physical and mental well-being is accessible as a toolbox at all times, but also referred to at relevant moments throughout the rest of the content.

Within each of the two thematic components SER and SSIP, four different modules include informational reading on ACE and ACE-related topics, as well as prompts for participants to actively reflect on what was learned in the readings and exercises to apply their knowledge and practice skills (see Table 1). The first module includes information about ACE in relationship with SER or SSIP respectively. Modules 2 and 3 contain information about the theoretical background of SER or SSIP respectively as well as information, writing tasks and exercises on the modification of possible cognitive biases. In module 4, the focus is on learning new skills and their implementation/transfer in daily life. Participants are encouraged to work through one module per week and to complete the exercises, which adds up to approximately one hour of engagement with the app per week.

**Table 1:** Outline of the components of the FACE self-help app

	**Introduction module** (always available) - Explanation of how the app works - Formulation of personal goals - Psychoeducation on prevalence, types, and consequences of ACE - Contact information on where to find additional help	
	**Toolbox with short exercises for daily life** Exercises and suggestions connected to - emotion regulation, i.e. to distraction, relaxation, encouragement, perspective and enjoyable activities (SER component) - social relationships, i.e. to personal boundaries, feeling at ease before, during and after social situations, social challenges and enjoyable activities (SSIP component)	
**Thematic Modules**	**SER Component**	**SSIP Component**
1. Psychoeducation and personal relevance of topics	- Importance of emotions for one’s life, thoughts, and behaviour - Emotion regulation and ACE	- Importance of relationships and reflection on status quo of personal relationships - Relationships and ACE
2. Promoting behavioural skills	- Emphasis on the active influencing of situations, behaviour and feelings to minimise negative and push positive emotions	- Emphasis on the identification and active promotion of social skills
3. Identification and resolving of dysfunctional cognitive patterns	- Identification of biased automatic perception and thought patterns, and how to counter them	- Identification of biased expectations towards social situations and interpretations of other people’s behaviour, and how to counter them
4. Promoting skills and long-term improvement	- Problem solving and the creation of favourable conditions for one’s emotional experience	- Dealing with difficult social situations and the building of positive social experiences - Learning to play to one’s social strengths
	**Final module/Farewell** - Summary and reflection of what has been learnt - Reiteration of contact information to find additional help - Tips on how to continue	

The FACE app was developed in an iterative co-design with emerging adults to deliver relevant interventions in an attractive, easy-to-use and low-threshold framework adapted for the target group. First, interviews with 29 emerging adults with a history of ACE were conducted to determine their expectations of a digital self-help intervention. Two focus groups accompanied in the development of the content of the app which included psychoeducative materials and exercises in various formats (text, audio, video). The app adapted content of different existing web-based self-help interventions such as the ECoWeb app for addressing social cognition and emotion regulation [67, 68], the SOLUS program for addressing loneliness and social activities [69], the HERMES program for problem-solving [70], the LEAVES program for emotion regulation, social relationships, and activities [71] and the REMOTION program for emotion regulation [72, 73]. The FACE app will be implemented as a progressive web app (PWA) and participants can access the PWA either from their smartphone or from regular internet browsers through a secure website, using a password-protected personal account.

#### Participants’ support and guidance

During the 10 weeks of the use of the FACE self-help programme, participants will receive weekly written feedback and support via an integrated messenger function - either from a psychologist or social worker of the School of Social Work at the University of Applied Sciences and Arts Northwestern Switzerland or from trained and supervised master students in Clinical Psychology of the University of Bern. This weekly support, which was short but individualised in terms of progress and focus, acknowledges and motivates participants in their engagement with the app, keeping emphasis on positive reassurance. It aims to improve adherence, provides a weekly structure, support for technical problems and the possibility to ask further questions. If needed, stabilising interventions via phone calls are offered by a psychologist within the FACE team who can also, if needed, provide support with seeking more comprehensive treatment through counselling services, psychotherapists or psychiatrists close to where the participants live. Reports of adverse events and negative effects will be collected.

### Statistical analyses

Analyses will be conducted according to the intention-to-treat paradigm. Additionally, we will conduct per-protocol analyses, including participants who complete baseline and post-intervention assessments and log into at least four modules defined as minimal therapeutic exposure. The significance level will be two-sided at *p* ≤ 0.05. To test the efficacy of the intervention in the intervention group compared to the control group, we will use linear mixed models. The differential effects of the SER and SSIP components will then be tested with interactions between the component and time. These analyses will model random slopes and intercepts for participants, test the fixed effects of the condition, as well as repeated assessments over time, using data from all participants. Linear mixed models have the advantage that they can account for missing values through maximum likelihood estimation [74]. Cohen’s d and f will be calculated as effect sizes. Furthermore, we will calculate Reliable Change Indexes [75].

Potential predictors and moderators of intervention outcome, such as gender or ACE severity will be analysed using multiple linear regressions. To analyse the mediation hypotheses and the longitudinal interplay of the variables, we will conduct path and structural equation analyses. Analyses will be conducted in SPSS, R and Mplus. There are no statistical stopping rules in this trial.

#### Sample size calculations

*Main effects*: We expect a moderate effect size of *f* > 0.25 for the comparison of the intervention and the waiting list control group and a small effect of *f* > 0.10 for the differential effects between SER and SSIP. Based on previous studies on web-based self-help interventions, we expect a correlation between the pre- and post-measurement of *r* = 0.6. Power analyses were conducted using G*Power [76] with a power of 0.8, an alpha error of 0.05. With three measurements, a sample size of 24 is required to detect a within-between interaction between the active intervention and the waitlist control group. A sample size of 162 is required to detect a small effect between the two active conditions and the waitlist control group.

*Intensive longitudinal data analysis*: Furthermore, the study combines data from the EMA with survey data in a multi-level model to investigate mechanisms and moderators of changes in well-being and resilience in the participants over the course of the study. Structural equation models including mediation analyses require a minimum sample of 241 for detecting a moderate effect (four latent and 25 observed variables [77]). This sample size will also allow accurate estimates of the regression coefficients, variance components and standard errors for multilevel analyses of the EMA data [78]. Sample size required for intensive longitudinal analysis is still a subject of ongoing research and it depends largely on (a) the effect size, (b) the number of measurements, and (c) the proportion of measures answered each day. Consequently, we aim to randomize up to 250 participants.

#### Data collection and analysis

Outcome and ecological momentary data will be assessed using online questionnaires programmed in the FACE self-help app. Data integrity is ensured through a variety of mechanisms, i.e. referential data rules, valid values, range checks, and consistency checks. In addition, data on the use of the modules are collected within the platform. All the data will be identified by a code that is not related to the participant’s identity. Servers are protected by high-end firewall systems and are located in data centres in the EU (Helsinki, Falkenstein) that comply with the GDPR and are ISO 27,001 certified. All the data will be handled confidentially and in addition to the technical administrators, only the researchers directly involved in the study have access to the data. Three audits and data monitoring will be performed by the person responsible for ethics and data security at the School of Social Work of the University of Applied Sciences and Arts Northwestern Switzerland.

#### Handling of missing data and dropout

Dropout is defined as participants who withdraw actively from the intervention after randomisation. All the other participants will receive an e-mail invitation to complete the post- and follow-up-questionnaires in any case. All dropout cases are part of the intent-to-treat sample, as they have been randomised and are included in the analyses. We will analyse the extent of missing data, explore the missing data patterns, and determine the type of missing data (Missing Completely at Random, Missing at Random, Not Missing at Random). Depending on the analysis, missing values will be replaced using multiple imputations. Sensitivity analyses will explore the impact of the imputation of missing values.

## Discussion

Adverse childhood experiences are common, cause substantial personal suffering and are a well-established transdiagnostic risk factor for various mental and physical health conditions throughout life. While primary prevention for reducing ACE is crucial, selective prevention aims to mitigate the harmful short- and long-term consequences of ACE. The guided FACE app for fostering resilience and well-being among emerging adults with a history of ACE targets self- and emotion regulation as well as social skills and biased social information processing. Thus, it targets risk processes instead of treating later symptoms of a disorder. Furthermore, it is delivered at an opportune time in life when emerging adults start to take control of their own lives and become independent of their parents. This stage in life offers a window of opportunity for recovery, but also holds a risk for the consolidation of problems and deterioration of trajectories. Thus, the FACE app has high potential for preventing and mitigating the adverse effects of ACE and thus reducing the need for more intensive psychotherapy for chronic dysfunctions later in life.

The FACE app was developed in an iterative co-design with emerging adults and delivers the interventions in an easy-to-use and low-threshold framework adapted for the target group. It offers psychoeducative materials and exercises in various formats (text, audio, video) on ACE and their impact and features a variety of interactive tools for use in everyday life. Additionally, participants receive one short personal feedback message a week to encourage the engagement with the app.

The primary aim of this study is to assess the efficacy of the app in enhancing resilience and well-being as well as other outcome variables in emerging adults with ACE. Additionally, the results will offer insights into the differential impact of the SER and SSIP components on fostering resilience and well-being and secondary outcomes such as self-efficacy for managing emotions, problem-solving, or fear of negative evaluation. The FACE intervention study is embedded in a three-wave epidemiological study to investigate emotion regulation and social information processing as mechanisms that link ACE with psychosocial functioning in emerging adulthood [50]. The epidemiological study based on a random population sample is used to identify emerging adults with a history of ACE who then are invited to take part in the intervention. Thus, the sample of the intervention study may include participants who would not have sought help on their own and who may be less self-selective than samples in intervention studies with a passive recruitment strategy. Moreover, the longitudinal epidemiological data also offer a broad basis for identifying predictors and moderators of treatment outcomes, such as the type and severity of ACE or previous help-seeking.

In addition to the epidemiological data, the retrospective self-report questionnaires for evaluating the app are complemented with EMA data on affective states, social interactions and coping strategies before and after the intervention as well as between the two components. The EMA data can detect context-dependent associations between emotion regulation, social information processing and psychosocial functioning in daily life. It provides real-life data for the evaluation of the app that is less prone to memory bias and can provide ecologically more valid insights into the effects of the app. Thus, by drawing on the longitudinal epidemiological and the EMA data, the FACE study extends the existing knowledge on self- and emotion regulation and social information processing as mechanisms for fostering resilience in emerging adulthood.

Limitations of the study are that self-help interventions have high dropout rates and that the effect sizes are lower than in blended counselling interventions [79, 80]. Furthermore, all the data from the participants are self-reported and thus subject to different types of bias, notably social desirability bias and non-response bias.

The findings from this study will inform the development of other interventions for coping with a history of ACE. It will also suggest improvements to the FACE app and evaluate its potential for large scale deployment as a low-threshold intervention to support emerging adults with a history of ACE in the transition to adulthood. This is particularly relevant in the context of current challenges with access to mental health services for emerging adults in Switzerland and elsewhere. A broadly accessible, and low-threshold self-help app such as the FACE app holds promise in complementing existing mental health support offers, potentially reducing the personal and societal harm associated with ACE.

## Data Availability

No datasets were generated or analysed during the current study.

## Abbreviations

ACE: Adverse childhood experiences
E-health: Digital self-help interventions
EMA: Ecological momentary assessments
M-health (/Mobile-health): Interventions delivered on smartphones (subcategory of E-health)
PWA: Progressive web app
SER: Self- and emotion regulation
SSIP: Social skills and social information processing
